# A rare case of orbital tuberculosis with cold abscess and frontal bone lesion

**DOI:** 10.11604/pamj.2020.37.167.21298

**Published:** 2020-10-15

**Authors:** Mahdia Touati, Assoumane Ibrahim, Kadre Alio Kadre Ousmane, Muneer Al-Zekri, Ibrahim Baaré, Abdelhalim Morsli

**Affiliations:** 1 Department of Neurosurgery, CHU Bab El Oued, Algiers, Algeria,; 2 Neurosurgery Department, Maradi Reference Hospital, Maradi, Niger,; 3 Department of Maxilla Facial Surgery, Reference Hospital of Niamey, Niamey, Niger,; 4 Ophthalmology Department, Maradi Reference Hospital, Maradi, Niger

**Keywords:** Orbital, tuberculosis, osteolytic

## Abstract

Tuberculosis is a multisystem infectious disease caused by Mycobacterium tuberculosis and a leading infectious cause of morbidity and mortality worldwide. Orbital tuberculosis is a rare form of extra pulmonary tuberculosis, even in endemic areas. It may involve the soft tissue, lacrymal gland, periosteum, or bones of the orbital wall and can extend to adjacent paranasal sinuses or intracranial cavities. The delay in diagnosis can be due to the fact that the clinical signs simulate any inflammatory disease. The diagnosis is usually based on tissue examination in histopathological evidence presenting as granulomatous lesion or presence of acid fast bacilli (AFB). The long term anti tuberculosis therapy is the effective treatment. Here we present the case of orbital tuberculosis on a young man operated in our department and who had a good outcome under anti tuberculosis drugs for 12 months.

## Introduction

Tuberculosis is a multisystem infectious disease caused by *Mycobacterium tuberculosis* and a leading infectious cause of morbidity and mortality worldwide. Orbital tuberculosis is a rare form of extra pulmonary tuberculosis, even in endemic areas. It may involve the soft tissue, lacrymal gland, periosteum, or bones of the orbital wall and can extend to adjacent paranasal sinuses or intracranial cavities [1, 2]. The delay in diagnosis can be due to the fact that the clinical signs simulate any inflammatory disease. The diagnosis is usually based on tissue examination in histopathological evidence presenting as granulomatous lesion or presence of acid-fast bacilli (AFB) [3]. The long-term anti tuberculosis therapy is the effective treatment. Here we present the case of orbital tuberculosis on a young man operated in our department and who had a good outcome under anti tuberculosis drugs during 12 months.

## Patient and observation

**Figure 1:**
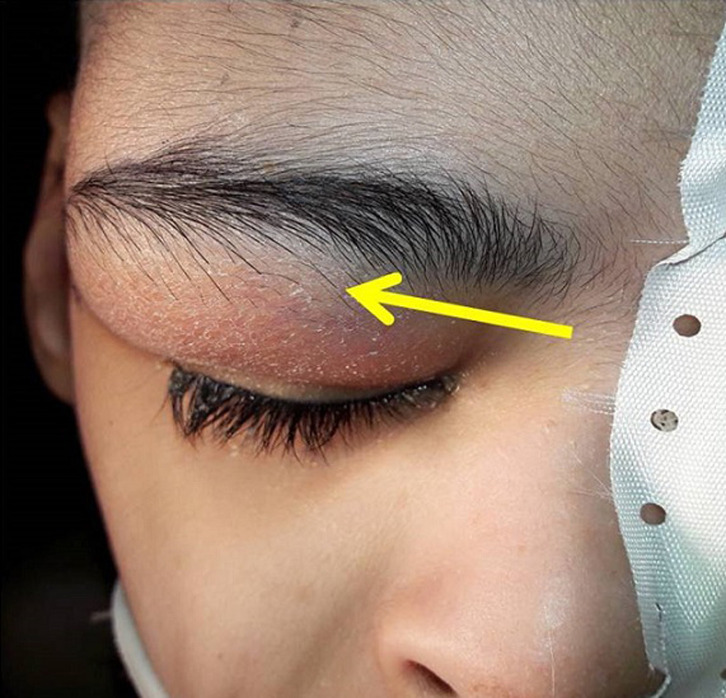
pre-operative image showing the eyelid tumefaction

**Figure 2:**
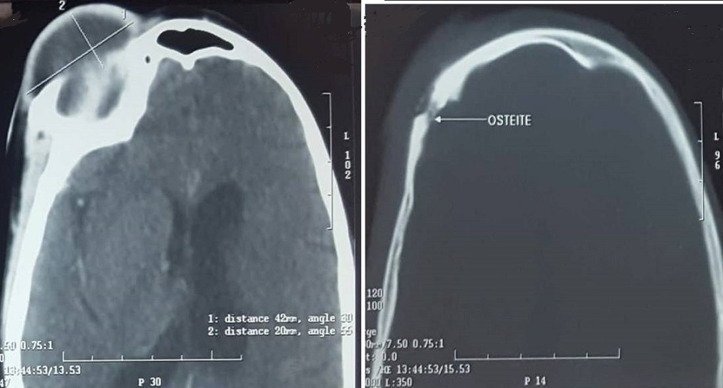
axial brain CT scan showing supra orbital collection and the frontal bone lytic lesion

**Figure 3:**
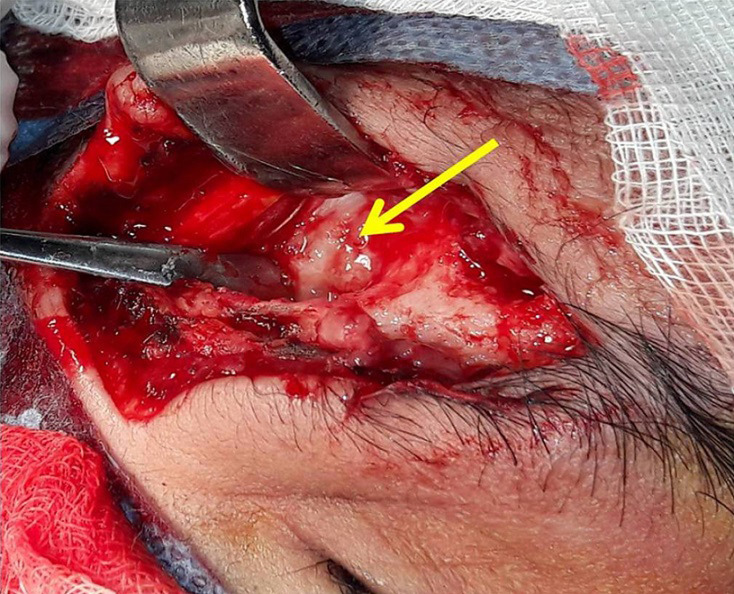
per-operative image showing osteolytic lesion (yellow arrow)

**Figure 4:**
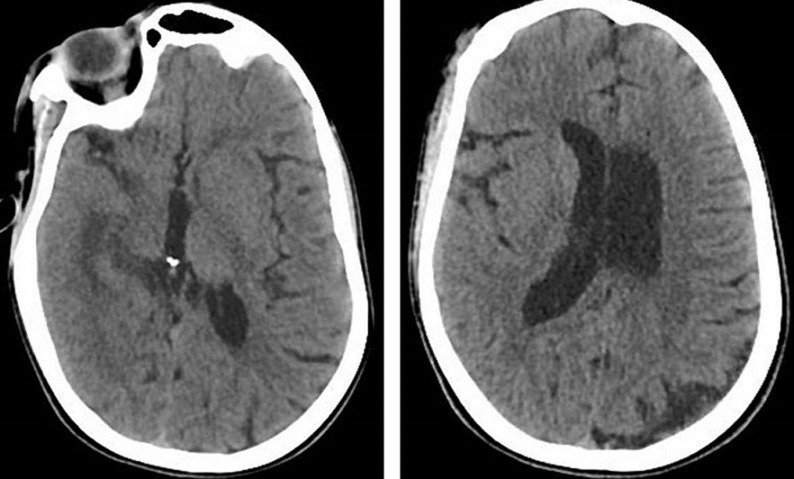
post-operative axial CT scan image; no collection and no lytic lesion

We report the case of a fifty years old patient with past medical history of cerebral palsy referred from ophthalmology department for right supra orbital swelling involving the eyelid. The beginning of signs is about four months by the onset of a sub cutaneous mass at the eyelid; he consulted at the ophthalmology department where after clinical examination a brain computed tomography (CT) scan was performed before his reference to the Neurosurgery Department. At the admission in our department the physical examination found a cachectic patient, complaining with fever in the evening and presenting a right supra orbital mass (Figure 1); the mass is non inflammatory, soft and painless. The brain CT scan objectified an osteolytic lesion of the orbital roof extended to frontal bone associated to an epidural lesion (Figure 2). We operated the patient under general anesthesia, we performed a skin incision through the right eyebrow; after the dissection of the subcutaneous tissues, we discovered and evacuated thick pus under the eyelid. We also discover an osteolytic lesion at the orbital roof extended to the frontal bone (Figure 3). We performed a biopsy of the bone lesion and the histological examination objectified a caseo follicular tuberculosis. The immediate post operative outcome was favorable with the disappearance of the collection. The patient was exited from the hospital 2 weeks after with a reference letter to infectious diseases department for anti tuberculosis treatment. The patient received the anti tuberculosis treatment for twelve months according to the national program. The control brain CT scan was performed after the completion of the treatment and there was no recurrence of the collection (Figure 4).

## Discussion

Tuberculosis (TB) is one of the top 10 causes of death worldwide according to the World Health Organization. In 2014, 9.6 million people fell ill with TB and 1.5 million died from the disease [4]. In 2018, 10 million people fell ill with TB, and 1.5 million died from the disease (including 251, 000 among people with HIV). The causative organism *Mycobacterium tuberculosis*, which is predominantly air-borne, affects the lung causing pulmonary TB. When TB is bacteriologically confirmed or clinically diagnosed in other parts of the body than the lung such as the abdomen, meninges, genitourinary tract, joints, bones, lymph nodes and skin it is classified as extrapulmonary tuberculosis (EPTB). The prevalence of EPTB among new and relapse TB cases globally in 2016 was 15% [5]. Tuberculosis of the orbit is extremely rare, even in places where tuberculosis is endemic [2, 6]. The literature on orbital tuberculosis management is dominated by case series and case report. In the 1980s, the human immunodeficiency virus (HIV) epidemic, homelessness, overcrowding, and immigration led to the resurgence of tuberculosis [7], Ajay K *et al.*[8] reported that in 2016 in India extra pulmonary TB comprises 10-15% of the total burden of TB. The incidence of extrapulmonary TB is on the rise because of human immunodeficiency virus (HIV)-TB correlation and emergence of drug resistance.

The index case presented with a sub cutaneous mass at the eyelid to the ophtalmlogy department, which is not specific sign of tuberculosis, the reason of misdiagnosis; but some signs are in favour of TB infection: bad general status, evening fever, mass without inflammation signs suggesting a cold abscess. The most common clinical manifestations of orbital tuberculosis are insidious and progressive unilateral proptosis, a cold, painless eyelid swelling, chemosis and conjunctival hyperemia; in some cases, there may be involvement of the ocular movements [9]. The differential diagnosis includes medial orbital subperiosteal abscess, complication of the sinusitis which requires surgical decompression when not responding to conservative treatment; orbital pseudotumor, lymphoma, and cavernous hemangioma [10]. In children, it is necessary to differentiate orbital tuberculosis from neuroblastoma (which causes orbital bone destruction), and dacryoadenitis (which is an inflammation of the lacrymal gland that can occur in cases of tuberculosis, syphilis, leprosy, cysticercosis, schistosomiasis, lymphoma, and sarcoid) [11, 12]. For Madge *et al*. [10] various manifestations of orbital tuberculosis (OTb) excluding ocular adnexal tuberculosis can be grouped under five clinical groups: 1) classical periostitis; 2) orbital soft tissue tuberculoma or cold abscess with no bony destruction; 3) OTb with evidence of bony involvement; 4) orbital spread from paranasal sinuses and 5) dacryoadenitis. Our patient could be classified in the third type because of the bony involvement. The orbital CT scan is useful to evaluate the bony involvement like in our case where this investigation objectified osteolytic lesion of the orbital roof extended to frontal bone. To confirm the diagnosis of TB we sent the biopsy of the bone lesion for histological examination which objectified a caseo follicular tuberculosis. The presence of AFB, characteristic histopathology, and growth of *Mycobacterium tuberculosis* from such a specimen remains the gold standard for diagnosis [10].

Standard anti-tubercular therapy (ATT) for 12-18 months completely resolves the lesion in most cases. Although medicine remains the mainstay of treatment, surgery is usually reserved for tissue diagnosis. Surgical approach depends on exact location of the lesion in the orbit and includes lateral orbitotomy, medial orbitotomy, and superior orbitotomy [13]. As in the index case we use ATT for 12 months started after the superior orbitotomy, this approach permitted us to drain the cold abscess and performed the biopsy of the involved bone.

## Conclusion

Orbital tuberculosis is a rare extra pulmonary presentation even in endemic region like Africa. In case of alteration of the general health status, fever in the evening and orbital mass in an endemic region of tuberculosis the diagnosis of orbital tuberculosis must be raised. Medical treatment (long term anti tuberculosis therapy) and surgery must be combined in case of cold abscess.

## References

[1] Shome D, Honavar SG, Vemuganti GK, Joseph J (2006). Orbital tuberculosis manifesting with enophthalmos and causing a diagnostic dilemma. Ophthalmic Plast Reconstr Surg.

[2] Aggarwal D, Suri A, Mahapatra AK (2002). Orbital tuberculosis with abscess. J Neuroophthalmol.

[3] Diyora B, Giri SA, Bhende B, Giri D, Kukreja S, Sharma A (2018). Orbital tuberculosis with intracranial extension. J Neurosci Rural Pract.

[4] WHO Tuberculosis: fact sheet.

[5] World Health Organization Global tuberculosis report 2017. Accessed on March 20.

[6] Dewan T, Sangal K, Premsagar IC, Vashishth S (2006). Orbital tuberculoma extending into the cranium. Ophthalmologica.

[7] Pillai S, Malone TJ, Abad JC (1995). Orbital tuberculosis. Ophthalmic Plast Reconstr Surg.

[8] Ajay K Verma, Anubhuti Singh, Kislay Kishore, Manoj Kumar Pandey, Surya Kant Orbital tuberculosis with involvement of the eyelid: An unusual presentation. The national Medical Journal of India. 2018;.

[9] Khalil M, Lindley S, Matouk E (1985). Tuberculosis of the orbit. Ophthalmology.

[10] Madge SN, Prabhakaran VC, Shome D, Kim U, Honavar S, Selva D (2008). Orbital tuberculosis: a review of the literature. Orbit.

[11] Narula MK, Chaudhary V, Baruah D, Kathuria M, Anand R (2010). Pictorial essay: orbital tuberculosis. Indian J Radiol Imaging.

[12] Van Assen S, Lutterman JA (2002). Tuberculous dacryoadenitis: a rare manifestation of tuberculosis. Neth J Med.

[13] Oliveira BF, Takay FC, Shida TM, Santo RM, Souza AC, Matayoshi S (2004). Orbital tuberculosis diagnosed by immunohistochemistry: case reports. Rev Inst Med Trop Sao Paulo.

